# Association of Electrocardiographic Signs of Right Ventricular Hypertrophy and Clot Localization in Chronic Thromboembolic Pulmonary Hypertension

**DOI:** 10.3390/jcm11030625

**Published:** 2022-01-26

**Authors:** Sylwia Sławek-Szmyt, Aleksander Araszkiewicz, Stanisław Jankiewicz, Anna Smukowska-Gorynia, Marek Grygier, Magdalena Janus, Maciej Lesiak, Tatiana Mularek-Kubzdela

**Affiliations:** 1st Department of Cardiology, Poznan University of Medical Sciences, 61-848 Poznan, Poland; aaraszkiewicz@interia.pl (A.A.); stanislaw.jankiewicz@skpp.edu.pl (S.J.); anna.smukowska-gorynia@skpp.edu.pl (A.S.-G.); marek.grygier@ump.edu.pl (M.G.); magdalena.janus@skpp.edu.pl (M.J.); maciej.lesiak@ump.edu.pl (M.L.); tatiana.mularek-kubzdela@skpp.edu.pl (T.M.-K.)

**Keywords:** chronic thromboembolic pulmonary hypertension, clot localization, electrocardiogram, right ventricular hypertrophy, diagnosis

## Abstract

The role of electrocardiography (ECG) in chronic thromboembolic pulmonary hypertension (CTEPH) diagnosis and prognosticating has not been yet established. We aimed to assess the relationships of the recommended ECG criteria of right ventricular hypertrophy (RVH) with clot localization in CTEPH patients. ECG patterns of RVH according to the American College of Cardiology Foundation were assessed in patients with newly diagnosed CTEPH. We enrolled 58 (45.3%) patients with proximal and 70 (54.7%) with distal CTEPH. Receiver-operating characteristics curves analysis indicated that the following ECG abnormalities predicted proximal CTEPH localization: R_V1_ > 6 mm—AUC 0.75 (CI: 0.66–0.84, *p* < 0.00001); S_V6_ > 3 mm—AUC 0.70 (CI: 0.60–0.79, *p* < 0.00001); S_I_ > R_I_ wave—AUC 0.67 (CI: 0.58–0.77, *p* = 0.0004); R_V1_:S_V1_ > 1.0—AUC 0.66 (CI: 0.56–0.76, *p* = 0.0009); R_V1_ peak > 0.035 s (QRS < 120 ms)—AUC 0.66 (CI: 0.56–0.75, *p* = 0.0016); R_V1_:S_V1_ > R_V3(V4)_:S_V3(V4)—_AUC-0.65 (CI: 0.54–0.75, *p* = 0.0081); R_aVR_ > 4 mm—AUC 0.62 (CI: 0.52–0.71, *p* = 0.002) and P_II_ > 2.5 mm—AUC 0.62 (CI: 0.52–0.72, *p* = 0.00162). Pulmonary vascular resistance significantly correlated with amplitudes of R_V1_ (r = 0.34, *p* = 0.008), S_V6_ (r = 0.53, *p* = 0.000027) and P_II_ (r = 0.44, *p* = 0.00007). In patients with CTEPH, only 8 out of 23 ECG RVH criteria were useful for differentiating between proximal and distal CTEPH localization and we found that R_V1_ and S_V6_ may contribute as potential discriminators.

## 1. Introduction

Chronic thromboembolic pulmonary hypertension (CTEPH) is a rare, progressive pulmonary vascular disease characterized by persistent obstruction of the pulmonary arteries by organized thromboembolic material. Previous studies reported a pooled incidence of CTEPH of 3.4% (95% CI 2.1–4.4%) after symptomatic pulmonary embolism [1,2,3]. The detailed pathophysiology of CTEPH remains unclear. Nevertheless, apart from the fibrotic transformation of pulmonary thrombi leading to nonhomogeneous vascular bed obstructions, the other undeniable mechanism is redistribution of the blood flow into the non-obstructed arteries and their remodeling, leading to an increase of the pulmonary arterial pressure and right ventricle failure development [1]. A pulmonary endarterectomy (PEA) is the gold standard of treatment for operable patients. However, some patients are ineligible for surgery due to distal localization of thromboembolic material and others who have undergone PEA suffered from persistent postoperative or recurrent pulmonary hypertension despite receiving optimal medical therapy [4]. In recent years, percutaneous balloon pulmonary angioplasty (BPA) has become a promising treatment modality for these CTEPH patients [5,6,7,8,9]. However, the efficacy of targeted medical treatment in CTEPH is limited [10,11].

Although, early CTEPH diagnosis significantly impacts the patient’s prognosis, it remains a challenge for clinicians [12]. Clinical symptoms are non-specific or absent in early CTEPH, whereas signs of right ventricle failure become evident in advanced stages. The reported median diagnostic delay of CTEPH diagnosis is 14 months in expert centers [2]. The diagnostic process of CTEPH is complex and requires pulmonary ventilation/perfusion scintigraphy, transthoracic echocardiography, and pulmonary angiography to be performed. However, there is an unfulfilled need to develop simple noninvasive tools to help clinicians identify patients with a high probability of CTEPH. Some previous studies revealed that in patients with pulmonary hypertension, the right heart overload is associated with the occurrence of the specific electrocardiographic changes [13,14]. There is a paucity of data regarding the role of noninvasive electrocardiography (ECG) in CTEPH diagnosis, management, and prognosticating.

Therefore, the aim of the present study was to evaluate relationships between electrocardiographic signs of right ventricular hypertrophy (RVH) and overload with hemodynamic as well as angiographic parameters in patients with newly diagnosed CTEPH. Moreover, the point of the present study was to differentiate, among the ECG markers of RVH/overload alterations, those better reflecting the pathologic remodeling of the pressure overloaded right ventricle that are not imposed by thromboembolic lesions and pulmonary vascular bed remodeling.

## 2. Materials and Methods

### 2.1. Study Population

The study group consisted of all consecutive patients with CTEPH diagnosed de novo in our department between November 2008 and November 2020. Patients were eligible to be included if they had performed invasive assessment and resting 12-lead ECG on the same day (to eliminate the impact of confounding factors). The diagnosis of CTEPH was defined as a mean pulmonary arterial pressure (mPAP) ≥ 25 mmHg and pulmonary artery wedge pressure (PAWP) ≤ 15 mmHg measured directly during right heart catheterization and a presence of perfusion defects on pulmonary angiography and/or computed tomography pulmonary angiography after at least three months of optimal anticoagulation therapy [15]. The other underlaying causes of chronic pulmonary hypertension were excluded by appropriate blood tests and imaging studies, including transthoracic echocardiography and cardiac magnetic resonance in accordance with the guidelines of the European Society of Cardiology (ESC) [15]. The operability evaluation of all patients was discussed by a multidisciplinary CTEPH team.

The recorded data included demographics, personal medical history, World Health Organization functional class (WHO-FC), six-minute walking distance (6MWD), N-terminal pro-B-type natriuretic peptide (NT-proBNP) concentration, and resting 12-lead ECG findings, along with echocardiographic, angiographic, and hemodynamic results. The study was in accordance with the Declaration of Helsinki and was approved by the bioethics committee of the Poznan University of Medical Sciences in Poznan, Poland (approval no. 725/16). Participation in the study did not influence routine diagnostic procedures or therapeutic decisions. Written informed consent was obtained from each patient included in the study.

### 2.2. Electrocardiography

A standard 12-lead electrocardiogram was performed in every patient during calm breathing in a supine position using Mortara ELI 250c electrocardiograph (Milwaukee, WI, USA). The ECG calibration was 25 mm/s and 10 mm/mV. Electrocardiographic patterns of RVH according to American College of Cardiology Foundation were assessed [16]. Details are presented in Table 1. Right bundle branch block (RBBB) was recognized in case of QRS duration ≥ 120 ms, with typical QRS morphology in lead *V*1 or *V*2 (rsr, rsR’, rSR’), and an *S* wave duration longer than the *R* wave duration or longer than 40 ms in lead I and lead *V*6 [17]. The control assessment was performed at three to six months after the interventional treatment of CTEPH (PEA or serial BPA procedures). Each ECG was assessed twice by two independent cardiologists (S.S.-S. and A.A.). Results were taken as the average of readings. We excluded ECGs which fulfilled any criterium of ischemia or left ventricular hypertrophy, or with significant arrhythmia, which may impact on the ECG curve analysis.

### 2.3. Right Heart Catheterization and Pulmonary Angiography

Right heart catheterization (RHC) was performed via the right internal jugular vein or right common femoral vein access using a flow-directed, balloon-tipped Swan–Ganz catheter (7F; Edwards Lifesciences, Irvine, CA, USA) in a supine position according to current guidelines [18]. The following pulmonary circulation parameters were directly measured or calculated: right atrial pressure (RAP, systolic, diastolic, and mean [mRAP]), right ventricular pressure (RVP, systolic, diastolic, and end-diastolic), pulmonary arterial pressure (PAP, systolic [sPAP], diastolic [dPAP] and mPAP), pulmonary arterial wedge pressure (PAWP), cardiac output (CO), cardiac index (CI), stroke volume (SV), pulmonary vascular resistance (PVR) and mixed venous saturation (SvO2). Cardiac output was evaluated using the thermodilution method. Pulmonary vascular resistance was determined as the difference between mPAP and PAWP divided by CO. The cut-off value for CTEPH recognition was mPAP equal to or greater than 25 mmHg, and PAWP less than 15 mmHg.

The conventional pulmonary angiography was conducted in accordance with current recommendations using a pigtail diagnostic catheter from the same venous access as in RHC [18]. A contrast agent (30–40 mL of amount and 10–25 mL/s of rate) was selectively administered into the right and left pulmonary artery through an automatic pump under a maximum pressure of 600–900 psi. Both arteries were selectively visualized in two oblique projections.

The computed tomography pulmonary angiography (CTPA) was also performed in each patient. CTPA was conducted on a multidetector-row CT to detect thromboembolic lesions, using automated bolus tracking in the main pulmonary artery and automated contrast medium injection with a flow rate of 3 mL per second, with a slice thickness of ≤1.5 mm and interval of 0.25 mm.

CTEPH was stratified as either proximal (lesions predominantly located in the main, lobar, and proximal segmental arteries) or distal (lesions distributed in distal segmental, subsegmental or more distal vessels with remodeling of microcirculation) based on the previously published San Diego intraoperative classification of the disease [19]. Although each side was assessed separately predominant location was included in this study.

### 2.4. Echocardiography

Transthoracic echocardiography was performed to qualitatively assess right ventricle strain parameters: RV free wall thickness, RV end-diastolic diameter, tricuspid annular plane systolic excursion (TAPSE), S’ wave and degree of tricuspid regurgitation.

### 2.5. Statistical Analysis

Patients’ characteristics are expressed as the frequency (percentage) for categorical variables and medians (interquartile ranges) for continuous variables. None of the variables had a normal distribution, as assessed using the Shapiro–Wilk test and Lilliefors test. Categorical variables were compared using the two-tailed Fisher’s exact test or χ^2^-test, or McNemar test (paired variables), and continuous variables were analyzed using the nonparametric Mann–Whitney U test, or Wilcoxon test (paired variables), as appropriate. The ANCOVA analyses were performed to control any potential confounding variables (no variable was identified as a significant confounder, and so no adjustment was needed). The Bonferroni correction was applied for the comparison of ECG parameters with the significance level set at a *p* value less than 0.0022 (0.05/23 = 0.0022) and, for the comparison of the RHC parameters, a *p* value less than 0.005 (0.05/10 = 0.005) was considered statistically significant. The intraobserver and interobserver agreements were estimated using the Kendall’s coefficient of concordance (W). The W- value was interpreted as follows: <0.20—poor agreement, 0.21–0.4—fair, 0.41–0.6—moderate, 0.61–0.8—good and 0.81–1.00—excellent agreement. Receiver-operating characteristics (ROC) analysis was performed to assess the discriminative capacity of ECG criteria between proximal and distal CTEPH cases. We calculated the area under the curve (AUC), as well as the sensitivity, specificity, accuracy, positive predictive values (PPV), negative predictive values (NPV), positive likelihood ratio (LR+), and negative likelihood ratio (LR-) of the currently recommended cut-off values for ECG RVH signs. Additionally, the cut-off values with the highest sensitivity and specificity were established for these criteria. Interactions between the ECG variables and hemodynamic data were tested using Spearman correlation tests. Statistical analysis was performed using Statistica version 13.7 (StatSoft, Inc., Tulsa, OK, USA).

## 3. Results

### 3.1. Study Population

The consecutive 128 patients with newly diagnosed CTEPH between November 2008 and November 2020 were enrolled in this study. Among the whole study group, 58 (45.3%) patients were diagnosed with proximal CTEPH, and 70 (54.7%) with distal CTEPH. The median age was 62 years (IQR: 52–70.5 years) and 50.8% of the patients were female. Patients with proximal CTEPH were significantly younger (59.5; IQR: 47–67 years vs. 67; IQR: 55–73 years, *p* = 0.0021) and more frequently suffered from symptomatic pulmonary embolism as compared with distal CTEPH (89.7% vs. 64.3%, *p* = 0.00087). There was no significant difference in patients’ WHO-FC between CTEPH groups. The majority of patients were in WHO-FC III (55.1% with proximal CTEPH and 60% with distal CTEPH, respectively) at initial assessment. CTEPH groups were also comparable regarding initial median 6MWD. The detailed characteristics of the study population are presented in Table 2.

### 3.2. Hemodynamic and Echocardiographic Results

We found significant differences in the median sPAP, mPAP, sRVP and PVR between the proximal and distal CTEPH groups. Both groups were comparable in terms of mRAP, PAWP, edRVP, dRVP, SVR, CO, CI and SvO2. In comparison to distal CTEPH, patients with proximal CTEPH had also significantly higher median RAA and TVPG. Other echocardiographic parameters were similar in both groups. Details are presented in Table 3.

### 3.3. Electrocardiographic Results

The overall interobserver agreement was very high (W = 0.83, *p* < 0.0001). The following ECG RVH parameters were significantly more frequent in the proximal CTEPH than in the distal CTEPH: RV1 > 6 mm, R_V1_: S_V1_ > 1.0, R_aVR_ > 4 mm, S_V6_ > 3 mm, R_V1_ peak > 0.035 s (QRS < 120 ms), S_I_ > R_I_ wave, R_V1_:S_V1_ > R_V3(V4)_:S_V3(V4)_, P_II_ > 2.5 mm. The detailed data are provided in Table 4.

### 3.4. Discriminatory Performance of Electrocardiography in CTEPH Localization

Details regarding the discriminatory performance of ECG RVH parameters are shown in Table 5.

**Figure 1:**
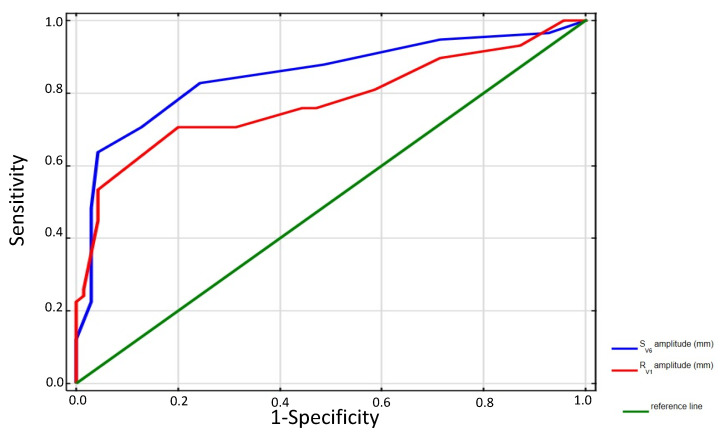
ROC analysis presenting the best cut-off value of R-wave amplitude in lead V1 and S-wave amplitude in V6. R_VI_ -best cut-off value 7 mm, AUC 0.78, 95% CI: 0.69–0.86, *p* < 0.0001; S_V6_ -best cut-off value 7 mm, AUC 0.85, 95% CI:0.78–0.92, *p* < 0.0001.

Receiver-operating characteristics curves analysis for the ECG parameters’ discriminative ability for CTEPH localization indicates R amplitude in V1 > 6 mm with a sensitivity of 70% and specificity of 80% along with S amplitude in V6 > 3 mm with a sensitivity of 88% and specificity of 51.4% as most important predictors. The currently recommended cut-off value of R wave amplitude in V1 was >6 mm predicted proximal CTEPH with AUC 0.753 (CI: 0.66–0.84; *p* < 0.00001). However, the best cut-off value of R wave amplitude in V1 was 7 mm with AUC 0.78 (CI: 0.69–0.86, *p* < 0.00001). The best cut-off value of S amplitude in V6 was 7 mm and it predicted proximal CTEPH with AUC 0.85 (0.78–0.92, *p* < 0.0001). Details are displayed in Figure 1.

### 3.5. Correlations between Electrocardiographic and Hemodynamic Data in Proximal CTEPH

The RVH ECG parameters were correlated with hemodynamic data—mPAP and PVR in patients with proximal CTEPH. Detailed results are shown in Table 6.

### 3.6. Changes in Electrocardiographic Parameters after Interventional Treatment of CTEPH

Changes in electrocardiographic parameters after PEA surgery or serial BPA procedures are shown in Table 7.

## 4. Discussion

Few previous studies have assessed the relationship between surface ECG and CTEPH for diagnostic and prognostic purposes [13,14,20,21,22]. To the best of our knowledge, this is the first study that aims to characterize RVH ECG signs in newly diagnosed CTEPH patients and to relate these parameters to the localization of thromboembolic material along with hemodynamic data. Although electrocardiography has traditionally been considered as method with an inadequate sensitivity and specificity for PH diagnosis, some previous studies showed that the sensitivity of ECG as a screening tool increased with PH severity [23,24]. It was recently demonstrated that the number of ECG abnormalities increased with the progression of PH [23]. Our study revealed that individual ECG variables pose a predictive ability for CTEPH localization diagnosis.

In the present study, the best discriminative ability between proximal and distal CTEPH had RV1 > 6 mm (AUC 0.75). The V1 lead is the only right-sided lead in the standard 12-lead ECG, and increased R amplitude in V1 corresponds to an enhanced net rightward depolarization. Previously, Al- Naamani et al. reported that RVH patterns in V1 appeared to have better PPV than the RVH criteria in V5 and V6 [25]. This could be explained by the fact that individual differences in R and S amplitudes in V5 and V6 are more probably related to the LV wall thickness than the RV thickness [25]. The RV1 amplitude was reported to be a marker of RV pressure overload. Previously, Kopeć et al. proved that the RV1 amplitude was a strong electrocardiographic predictor of RV mass in patients with pulmonary hypertension (r = 0.71, *p* = 0.0001). The authors also proposed the new cut-off value of RV1 amplitude for RVH prediction >3 mm [20]. Our study revealed that the R amplitude >7 mm in V1 possessed a better discriminative ability between the proximal and distal CTEPH. We demonstrated positive correlation between the RV1 amplitude and mPAP (r = 0.34, *p* = 0.008), as well as PVR, in the proximal CTEPH group (r = 0.29, *p* = 0.0115). Waligóra et al. also documented that the decrease of RV1 amplitude after targeted treatment implementation correlated with the reduction of mPAP (r = 0.33, *p* = 0.002) and PVR (0.21, *p* = 0.05) in a group of 80 patients with PAH and CTEPH [21]. Interestingly, Ghio et al. showed a significant reduction of RV1 amplitude in CTEPH patients one month after PEA in parallel with the rapid and sustained improvement in right heart hemodynamics, which is line with our results [14].

In the present study, the ECG pattern focusing on R/S ratio in the lead V1 also had a predictive ability. R_V1_/S_V1_ ratio ≧ 1 was significantly more frequent in the proximal CTEPH group than in the distal CTEPH group and significantly improved in both CTEPH groups after interventional treatment. However, the results of prior studies regarding the reduction of the R_V1_/S_V1_ ratio after BPA treatment are inconsistent. Piłka et al. reported a significant improvement of the R_V1_/S_V1_ ratio (*p* = 0.046) after series of BPA, but other studies showed that although a significant reduction of the RV1 amplitude after BPA was achieved, the R_V1_/S_V1_ ratio did not change significantly [21,25,26]. Moreover, Nishiyama indicated that the R_V1_/S_V1_ ratio ≧ 1 was the most frequent RVH marker after serial BPA procedures. [26]. We have demonstrated a significant reduction of the R_V1_/S_V1_ ratio after interventional treatment (PEA or BPA) in patients with proximal disease as well as after serial BPA procedures in distal disease.

The R_V1_:S_V1_ > R_V3(V4)_:S_V3(V4)_ ratio and S_I_ > R_I_ ratio, although supportive criterion of RVH were frequent ECG signs in both CTEPH groups in the current study. Both RVH markers significantly improved after interventional treatment (PEA or BPA) in patients with proximal disease, as well as after serial BPA procedures in distal disease. Our results contrast with previous studies that have shown no difference in the R_V1_:S_V1_ > R_V3(V4)_:S_V3(V4)_ ratio and S_I_ > R_I_ ratio, despite hemodynamic improvement in patients with inoperable CTEPH or pulmonary arterial hypertension (PAH) [21]. However, we assessed this parameter for the first time in newly diagnosed CTEPH and further studies are needed to evaluate the significance of these parameters.

We demonstrated that the S-wave amplitude in lead V6 was the second most frequent marker of RVH in patients with proximal and distal CTEPH but was significantly more common and had a higher median amplitude in proximal disease. SV6 had also discriminated satisfactorily between proximal and distal CTEPH (AUC 0.70). Nonetheless, the cut-off value of Sv6 amplitude with the highest discriminatory ability was 7 mm (AUC 0.85). It was reported that the amplitude of the S and R waves in lead V6 referred to a clockwise rotation of the heart [26]. Furthermore, we found significant correlations between the SV6 amplitude and mPAP (r = 0.53, *p* < 0.0001), and with the PVR (r = 0.4, *p* = 0.0024). Nishiyama et al. also showed significant correlation between the change in mPAP and SV6 amplitude reduction after BPA treatment in CTEPH patients [26]. Yokokawa et al. demonstrated a significant reduction of SV6 amplitude after BPA in a group of 19 patients with CTEPH (53% vs. 11%, *p* = 0.005, respectively) [27]. The present study also showed a significant reduction of the median SV6 amplitude after interventional treatment (PEA or BPA) in patients with proximal disease, as well as after serial BPA procedures in distal disease. Moreover, Kanemoto revealed that a SV6 of at least 0.7 mV indicated CI of less than 2.8 L/min/m^2^ in patients with primary PH with a sensitivity of 82% and a specificity of 86%, respectively [28].

It was also reported that P pulmonale (P wave amplitude >0.25 mV in lead II) is an ominous sign of poor prognosis [29]. In the current study the presence of P pulmonale discriminated CTEPH localization (AUC 0.621). P_II_ amplitude increases as a result a significant retrograde atrial overload due to progressive RVH-associated diastolic dysfunction, and RV dilatation-associated tricuspid regurgitation in patients with pulmonary hypertension. It was reported that in patients with CTEPH the P_II_ amplitude correlated with the PVR value [30]. The results of the present study are in line with previous data. We also found significant positive correlations between the proximal CTEPH and PVR value (r = 0.4, *p* = 0.002). Piłka et al. showed that the P_II_ amplitude correlated also with the change of PVR following BPA treatment (r = 0.44) [22]. Ghio et al. demonstrated a significant decrease in P_II_ amplitude within one month after pulmonary endarterectomy [14]. Moreover, we found a significant positive correlation between P_II_ amplitude and mPAP (r = 0.44, *p* = 0.00007). Several previous studies regarding PAH also proved the importance of the P-wave amplitude in revealing hemodynamic improvement in the mPAP and CI following the application of targeted pharmacotherapy [19,29]. Cheng et al. reported also that P_II_ was the independent predictor of all-cause mortality in patients with PAH (hazard ratio:1.555, *p* = 0.033) [31].

It was previously shown that the R wave amplitude in lead aVR was an independent predictor of all-cause mortality in patients with PAH, but there is a lack of data regarding its role in CTEPH [31]. Our study indicated significant differences between proximal and distal disease in the amplitude of the R wave in the aVR lead. Moreover, there was also a significant reduction of the median R amplitude after interventional treatment (PEA or BPA) in patients with proximal disease. This is in contrary to Piłka et al. study that noted no improvement in the R wave in aVR amplitude after BPA treatment [22].

From the non-voltage criteria, only the ventricular activation time in the lead V1, which expresses the depolarization interval time of ventricular depolarization from the endocardium to the epicardium discriminated between the proximal and distal CTPEH. In the present study, the RV1 peak > 0.035 s (QRS < 120 ms) presented discriminative ability (AUC 0.655) for CTEPH localization. Recently, Asano et al. showed that the QRS duration correlated with the area of fibrosis in the RV [32]. Similarly, Kopeć et al. also revealed the utility of the ventricular activation time in V1 in RVH diagnosis in PAH (AUC 0.83). Moreover, those researchers found that the ventricular activation time correlated with the RV mass (r = 0.54, *p* = 0.01) as well as the RV volume (r = 0.64, *p* = 0.002) [20]. The improvement in ventricular depolarization time after interventional treatment (PEA or BPA) in patients with proximal disease, as well as after serial BPA procedures in our study support these findings.

Some previous studies evaluated the correlations between ECG abnormalities and hemodynamic data in PH patients with and without RBBB. Nonetheless, the presence of RBBB influenced on several changes in ECG and, therefore, we analyzed separately patients with RBBB and without RBBB. Our results refer to patients without RBBB and, consequently, the specific ECG RVH patterns according to the AHA/ACCF/HRS are not useful to differentiate between the CTEPH group in patients with RBBB.

This study is burdened by several limitations. The study included a relatively small number of patients from a single center, which results from the low prevalence of the CTEPH entity. There was no control group in this study. We cannot also exclude selection bias due to the nature of the study design. There is a possibility that some ECG criteria of RVH would appear as statistically significant in a larger study group. This study is limited somewhat by a lack of recorded data for some variables that would be of interest at present, including a lack of initial ECG data for all the patients from the period of the acute pulmonary embolism episode. Moreover, no attempt was made to determine whose ECG findings were novel or preexisting after pulmonary embolism. An important study limitation is the assessment of the RV morphology and function was performed only by two-dimensional echocardiography; however magnetic resonance imaging would be more accurate in this setting. Furthermore, the timespan of study group enrollment was long and during this time the treatment approach for patients with CTEPH significantly improved, which could affect the follow-up outcome. The study is also limited by a lack of patients’ long-term follow-up results. In addition, in the present study we were unable to distinguish which specific ECG abnormalities are predominantly related to the thrombus burden or thickening and fibrosis of the RV free wall, or RV dilatation, or ischemia and injury of RV. Finally, ECG is currently recommended as an additional diagnostic modality for CTEPH screening, giving priority to imaging tests and to the right heart catheterization in the diagnosing of the CTEPH. Nonetheless, ECG is useful at an early stage of the CTEPH diagnostic workup and might be potentially included in a screening algorithm as a first test for the identification of patients at a high risk of CTEPH.

## 5. Conclusions

In patients with CTEPH, only 8 out of 23 ECG criteria were useful for differentiating between proximal and distal thromboembolic material localization. R-wave amplitude in V1 and S-wave in V6 had the highest discriminatory performance. These ECG patterns were in accordance with a hemodynamic data (mPAP and PVR values) which enables their potential future use in CTEPH diagnosis making process. However, the detailed clinical significance and contribution of these findings to the diagnostic and management algorithm of CTEPH requires further evaluation and validation.

## 6. Patents

This section is not mandatory but may be added if there are patents resulting from the work reported in this manuscript.

## Figures and Tables

**Table 1:** Electrocardiographic criteria of right ventricular hypertrophy.

ECG Sign	Definition—Amplitude
R-wave in lead V1	>6 mm
R-wave in lead V1 + S-wave in lead V5 (V6)	>10.5 mm
R-wave: S-wave ratio in lead V1	>1.0
R-wave in lead aVR	>4 mm
R-wave in lead V5 (V6)	<3 mm
S-wave in V1	<2 mm
S-wave in V5	>10 mm
S-wave in V6	>3 mm
R-wave: S-wave ratio in lead V5	<0.75
R-wave: S-wave ratio in lead V6	<0.4
R-wave: S-wave ratio in lead V5 to R-wave: S-wave ratio in lead V1	<0.04
(R-wave in lead I + S-wave in lead III) minus (S-wave in lead I + R-wave in lead III)	<15 mm
R-wave peak in lead V1 (QRS < 120 ms)	>0.035 s
QR complex in lead V1	present
Supportive criteria	
RSR’ complex in lead V1 (QRS duration > 0.12 sec)	present
S wave > R wave in lead I, II, III	present
S-wave in lead I and Q-wave in lead III	present
Negative T-wave in leads V1–V4	present
R-wave: S-wave ratio in lead V1 > R-wave: S-wave ratio in lead V3 (V4)	present
P-wave in lead II	>2.5 mm
Right ventricular strain	
ST-T segment depression by at least 1 mm in leads V1–V3	present
ST-T segment depression by at least 1 mm in leads: II, III, aVF	present
negative T wave in leads: II, III, aVF	present

**Table 2:** Demographic and clinical characteristics of the study group (number, percentage).

Parameter	All (*n*, (%)	Proximal CTEPH (*n* = 58)	Distal CTEPH (*n* = 70)	*p* Value
Age (years), median (IQR)	62 (52–70.5)	59.5 (47–67)	67 (55–73)	0.0021
Sex				0.053
(female/ male)	65 (50.8)/	35 (60.3)/	30 (42.9)/	
	63 (49.2)	23 (39.7)	40 (57.1)	
BMI (kg/m^2^)	29 (25.5–33.1)	27.7 (24.2–32.3)	29.5 (26–33.3)	0.64
BSA	1.9 (1.7–2.1)	2.0 (1.7–2.2)	1.9 (1.7–2.1)	0.013
WHO FC				0.22
I	-	-	-	
II	28 (21.9%)	11 (19%)	17 (24.3%)	
III	74 (57.8%)	32 (55.1%)	42 (60%)	
IV	26 (20.3%)	15 (25.9%)	11 (15.7%)	
Previous symptomatic pulmonary embolism	97 (75.8%)	52 (89.7%)	45 (64.3%)	0.00087
Chronic obstructive pulmonary disease	16 (12.5%)	4 (6.9%)	12 (17.1%)	0.1
Diabetes mellitus	20 (15.6%)	6 (10.3%)	14 (20%)	0.15
Systemic arterial hypertension	85 (66.4%)	37 (63.8%)	48 (68.6%)	0.58
Known thrombophilia	6 (4.7%)	4 (6.9%)	2 (2.9%)	0.29
Chronic renal insufficiency	30 (23.4%)	13 (22.4%)	17 (24.3%)	0.83
Anticoagulation therapy				
DOAC	80 (64.5%)	34 (58.6%)	46 (67.7%)	0.35
VKA	42 (33.9%)	22 (37.9%)	20 (30.3%)	0.45
6MWD (m), median (IQR)	310 (250–390)	298.5 (260–405)	320 (240–390)	0.47
NT-proBNP (pg/mL), median (IQR)	1020 (286–2177)	1038 (355–1977)	1002 (286–3436)	0.59

Abbreviations: BMI—body mass index; BSA—body surface area; DOAC—direct oral anticoagulant; IQR—interquartile range; NT-proBNP—N-terminal brain natriuretic propeptide; WHO-FC—World Health Organization Functional Class; VKA—vitamin K antagonist; 6MWD—6-min walking distance.

**Table 3:** Comparison of hemodynamic and echocardiographic data between proximal and distal CTEPH (median, interquartile ranges).

Parameter	All *n* = 128	Proximal CTEPH (*n* = 58)	Distal CTEPH (*n* = 70)	*p* Value
Hemodynamic data				
sSAP (mmHg)	139.5 (128–155)	134 (125–142)	144 (128–166)	0.001
dSAP (mmHg)	86.5 (80–92)	76 (56–95)	87 (82–92)	0.34
mRAP (mmHg)	7 (5–11.5)	6.5 (5–13)	8 (5–10)	0.97
sRVP (mmHg)	78.5 (63.5–89)	82 (70–92)	70.5 (62–83)	0.0018
dRVP (mmHg)	5.8 (2–8)	3 (5–6)	2 (5–8)	0.62
edRVP (mmHg)	11 (8–16)	11.5 (8–16)	11 (8–15)	0.62
sPAP (mmHg)	78.5 (63.5–89)	82 (70–92)	70.5 (62–83)	0.0018
mPAP (mmHg)	45 (40–52)	48 (44–57)	43 (39–50)	0.0004
PAWP (mmHg)	7 (10–12)	10 (7–12)	10 (7.5–10)	0.49
PVR (Wood units)	6.9 (5.4–8.6)	7.3 (5.6–10.9)	6.2 (4.6–8.1)	0.0038
SVR (Wood units)	16.4 (13.8–20.1)	16.7 (14.4–20.1)	16.3 (13.3–19.9)	0.8
CO (L/min)	5.8 (4.65–6.6)	5.8 (5.0–6.6)	5.8 (4.6–6.6)	0.8
CI (L/min/m^2^)	2.9 (2.7–3.5)	2.9 (2.7–4.5)	3.1 (2.4–3.6)	0.64
SV (mL)	76.5 (63–98)	81 (66–101)	72 (60–98)	0.56
SvO2 (%)	67.8	68.7 (64.5–72.8)	67.4 (62.6–74)	0.7
SaO_2_ (%)	92.3 (91–94)	92.7 (91.4–94)	92.2 (90.9–93.9)	0.35
Echocardiographic data				
RAA (cm^2^)	24.1 (21–32)	27 (21–36)	23 (20–28)	0.0278
RV free wall thickness (mm)	5.5 (4.5–6)	5.9 (4.8–6)	5.3 (4.5–5.8)	0.3
RV end-diastolic diameter (4 chamber) (mm)	47 (43–53)	47 (43–53)	45 (42–52)	0.39
TAPSE (mm)	19 (15–24)	19 (14–22)	19 (16–25)	0.17
TRV max (m/s)	4.4 (3.9–4.7)	4.5 (4.2–4.8)	4.4 (3.6–4.6)	0.06
TVPG (mmHg)	84 (60–95)	90 (70–95)	76 (55–90)	0.014
Pulmonary trunk (mm)	31 (26–36)	32 (28–37)	29 (25–35)	0.24
S’ (cm/s)	12 (9–15)	11 (9–14)	12.5 (9–15)	0.095

Abbreviations: CI—cardiac index; CO—cardiac output; CTEPH—chronic thromboembolic pulmonary hypertension; dRVP—diastolic right ventricular pressure; dSAP—diastolic systemic arterial pressure; edRVP—end-diastolic ventricular pressure; mPAP—mean pulmonary arterial pressure; mRAP—mean right atrial pressure; PAWP—pulmonary artery wedge pressure; PVR—pulmonary vascular resistance; RAA—right atrium area; RV—right ventricle; sRVP—systolic right ventricular pressure; sPAP—systolic pulmonary arterial pressure; SaO2- arterial blood saturation; sSAP—systolic systemic arterial pressure; SV—stroke volume; SVR—systemic vascular resistance, SvO2—and mixed venous saturation; TAPSE—tricuspid annular plane systolic excursion; TRV—tricuspid regurgitation velocity; TVPG—tricuspid valve pressure gradient.

**Table 4:** Comparison of the frequency of electrocardiographic parameters between proximal and distal CTEPH (number, percentages, or median, interquartile ranges).

Parameter	All	Proximal CTEPH (*n* = 58)	Distal CTEPH (*n* = 70)	*p* Value
HR (bpm), median, (IQR)	78 (70–90)	80 (72–98)	76 (67–82)	0.64
Sinus tachycardia, n (%)	18 (14)	11 (19)	7 (10)	0.2
Axis				0.61
Normal, n (%)	62 (48.4)	28 (48.3)	34 (48.6)	
Right axis deviation, n (%)	58 (45.3)	24 (41.4)	34 (48.6)	
Left axis deviation, n (%)	8 (6.3)	6 (10.3)	2 (2.9)	
R_V1,_ median, (IQR)	5 (3–8)	8 (5–9.5)	4 (2–6)	<0.0001
R_V1_ > 6 mm, n (%)	55 (43)	41 (70.7)	14 (20)	<0.0001
R_V1_ + S_V5 (V6)_, median, (IQR)	11 (8–13.8)	12 (9–18)	9.5 (7–12)	0.002
R_V1_ + S_V5 (V6)_ > 10.5 mm, n (%)	70 (54.7)	40 (69)	30 (42.9)	0.004
R_V1_:S_V1_ > 1.0, n (%)	78 (60.9)	45 (77.6)	33 (47.1)	0.0005
R_aVR_ (mm), median, (IQR)	3 (2–4)	4.5 (2.5–5)	2 (1–3)	<0.0001
R_aVR_ > 4 mm, n (%)	36 (28.1)	31 (53.5)	5 (7.1)	<0.0001
R_V5 (V6)_ (mm), median, (IQR)	6 (5–8)	6 (5–8)	6 (5–8)	0.69
R_V5 (V6)_ < 3 mm, n (%)	6 (5)	2 (3)	4 (6)	0.69
S_V1_(mm), median, (IQR)	1 (0.8–4)	1 (1–2)	1 (0.8–5)	0.28
S_V1_ < 2 mm, n (%)	69 (53.9)	39 (67.2)	30 (42.9)	0.024
S_V5_ > 10 mm, median, (IQR)	5 (3–7.5)	6 (5–10)	4 (3–6.5)	0.0054
S_V5_ >10 mm, n (%)	15 (12)	11 (19)	4 (6)	0.027
S_V6_ (mm), median, (IQR)	5 (3–7)	7 (5–8)	3 (2–4)	<0.0001
S_V6_ > 3 mm n (%)	85 (66)	51 (88)	34 (48.6)	<0.0001
R_V5_:S_V5_ < 0.7, n (%)	19 (15)	10 (17)	9 (13)	0.62
R_V6_:S_V6_ < 0.4, n (%)	6 (5)	2 (3)	4 (6)	0.69
R_V5_:S_V5_ to R_V1_:S_V1_ < 0.04	3 (2.34)	3 (5.2)	0	0.9
(R I + S III) − (S I + RIII) < 15 mm, n, (%)	101 (78.9)	42 (72.4)	59 (84.3)	0.128
R_V1_ peak > 0.035 s (QRS < 120 ms), n, (%)	60 (46.9)	37 (63.8)	23 (32.9)	0.0007
qR_V1,_ n (%)	9 (7)	5 (9)	4 (6)	0.73
Supportive criteria				
RSR’ complex in lead V1 (QRS duration > 0.12 s), n (%)	9 (7)	5 (9)	4 (6)	0.73
S_I_ > R_I_ wave, n (%)	62 (48.4)	39 (67.2)	23 (32.9)	0.00017
S_II_ > R_II_ wave, n (%)	31 (24.2)	19 (32.8)	12 (17.1)	0.06
S_III_ > R_III_, n (%)	24 (18.8)	15 (25.9)	9 (12.9)	0.07
S_I_Q_III,_ n (%)	83 (65)	41 (71)	42 (60)	0.26
Negative T_V1–V4_, n (%)	85 (66)	41 (71)	44 (63)	0.45
R_V1_:S_V1_ > R_V3(V4)_:S_V3(V4)_, n (%)	76 (75.3)	45 (90)	31 (60.8)	0.001
P_II_ > 2.5 mm, median, (IQR)	2 (1–2.5)	2.0 (1.5–3.0)	2.0 (0.5–2)	0.0008
P_II_ > 2.5 mm, n (%)	30 (24)	19 (34)	11 (16)	0.02
RV strain				
ST-T segment depression V1–V3, n (%)	87 (68)	43 (74)	44 (43)	0.18
ST-T segment depression II, III, aVF, n (%)	65 (51)	32 (55)	33 (47)	0.38
negative T II, III, aVF, n (%)	65 (51)	32 (55)	33 (47)	0.38

Abbreviations: CTEPH—chronic thromboembolic pulmonary hypertension; HR—heart rate; IQR—interquartile range; RV—right ventricle.

**Table 5:** ROC analysis for the ECG parameters’ discriminative ability between proximal and distal CTEPH.

ECG Signs	AUC	95% CI	*p* Value	Sensitivity (%)	Specificity (%)	Accuracy (%)	PPV (%)	NPV (%)	LR+	LR−
R_V1_ > 6 mm	0.75	0.66–0.84	<0.00001	70	80	75.8	74.5	76.7	3.5	0.37
R_V1_:S_V1_ > 1.0	0.66	0.57–0.76	0.0009	79.3	52.9	64.8	58.2	75.5	1.68	0.39
R_aVR_ > 4 mm	0.62	0.52–0.71	0.02	65.5	74.3	61	71	70.7	1.7	0.75
S_V6_ > 3 mm	0.70	0.60–0.79	<0.00001	88.0	51.4	68	60	83.7	1.8	0.24
R_V1_ peak > 0.035 s (QRS < 120 ms)	0.69	0.59–0.79	0.0002	80.4	58.2	68.9	61.7	69.1	0.31	0.34
S_I_ > R_I_	0.67	0.58–0.77	0.0004	67.2	67.1	67.2	62.9	71.2	2.0	0.49
R_V1_:S_V1_ > R_V3(V4)_:S_V3(V4)_	0.65	0.54–0.75	0.0081	90	39.2	64.4	59.2	80	1.49	2.55
P_II_ > 2.5 mm	0.62	0.52–0.72	0.0162	41.4	82.9	64.1	66.7	63	2.41	0.71

Abbreviations: AUC—area under the curve; CI—confidence interval; CTEPH—chronic thromboembolic pulmonary hypertension; ECG—electrocardiogram; LR+—positive likelihood ratio; LR−—negative likelihood ratio NPV—negative predictive value, PPV—positive predictive value; ROC—receiver-operator characteristic.

**Table 6:** Correlations between ECG criteria of RVH with mPAP and PVR in proximal CTEPH.

	Proximal CTEPH			
ECG Signs	mPAP		PVR	
	r	*p*	r	*p*
R_V1_	0.34	0.008	0.29	0.015
R_V1_:S_V1_	0.11	0.42	0.24	0.07
R_aVR_	0.21	0.076	0.25	0.044
S_V6_	0.53	0.000027	0.4	0.0024
R_V1_ peak (QRS < 120 ms)	0.24	0.075	−0.06	0.66
S_I:_ R_I_	0.31	0.009	0.2	0.093
R_V1_:S_V1/_R_V3(V4)_:S_V3(V4)_	0.31	0.026	−0.16	0.26
P_II_	0.44	0.00007	0.4	0.002

Abbreviations: CTEPH—chronic thromboembolic pulmonary hypertension; ECG—electrocardiogram; mPAP—mean pulmonary arterial pressure, PVR—pulmonary vascular resistance.

**Table 7:** Changes in electrocardiographic parameters 3–6 months after interventional treatment of CTEPH.

ECG Sign	Proximal CTEPH		*p* Value	Distal CTEPH		*p* Value
	On Diagnosis (*n* = 58)	3–6 Months after PEA (*n* = 36)/BPA Treatment (*n* = 12)		On Diagnosis (*n*= 70)	3–6 Months after BPA Treatment (*n* = 45)	
R_V1_ > 6 mm, median, (IQR)	8 (5–9.5)	4 (1–8)	<0.00001	4 (2–6)	4.5 (2–5)	<0.00001
R_V1_ > 6 mm, n (%)	41 (70.7)	15 (31.2)	<0.0001	14 (20)	5 (11.1)	0.0078
R_V1_:S_V1_ >1.0	45 (77.6)	19 (39.6)	<0.0001	33 (47.1)	10 (22.2)	<0.0001
R_aVR_ > 4 mm, median, (IQR)	4.5 (2.5–5)	5.2 (4.7–6)	<0.0044	2 (1–3)	-	-
R_aVR_ > 4 mm, n, (%)	31 (53.5)	12 (25)	<0.0001	5 (7.1)	-	-
S_V6_ (mm), median, (IQR)	7 (5–8)	2 (2–2.5)	<0.0001	3 (2–4)	2 (1–2)	0.0025
S_V6_ > 3 mm n (%)	51 (88)	9 (18.8)	<0.0001	34 (48.6)	6 (13.3)	<0.0001
R_V1_ peak > 0.035 s (QRS < 120 ms)	37 (63.8)	16 (33.3)	<0.0001	23 (32.9)	6 (13.3)	0.0001
S_I_ > R_I_, n (%)	39 (67.2)	11 (22.9)	<0.0001	23 (32.9)	11 (24.4)	0.0015
R_V1_:S_V1_ > R_V3(V4)_:S_V3(V4)_	45 (90)	14 (29.2)	<0.0001	31 (60.8)	11 (24.4)	<0.0001
P_II_ > 2.5 mm, median, (IQR)	2.0 (1.5–3.0)	2.0 (1.5–2.0)	0.0002	2.0 (0.5–2)	2 (0.5–2)	0.14
P_II_ > 2.5 mm, n, (%)	19 (34)	8 (16.7)	0.0026	11 (16)	8 (17.8)	0.25

Abbreviations: BPA—balloon pulmonary angioplasty; CTEPH—chronic thromboembolic pulmonary hypertension; IQR—interquartile range; PEA—pulmonary endarterectomy.

## Data Availability

The data that support the findings of this study are available from the corresponding author, upon reasonable request.
